# Super-resolution imaging of fluorescently labeled, endogenous RNA Polymerase II in living cells with CRISPR/Cas9-mediated gene editing

**DOI:** 10.1038/srep35949

**Published:** 2016-10-26

**Authors:** Won-Ki Cho, Namrata Jayanth, Susan Mullen, Tzer Han Tan, Yoon J. Jung, Ibrahim I. Cissé

**Affiliations:** 1 Department of Physics, Massachusetts Institute of Technology, 77 Massachusetts Avenue, Cambridge, MA 02139, USA

## Abstract

Live cell imaging of mammalian RNA polymerase II (Pol II) has previously relied on random insertions of exogenous, mutant Pol II coupled with the degradation of endogenous Pol II using a toxin, α-amanitin. Therefore, it has been unclear whether over-expression of labeled Pol II under an exogenous promoter may have played a role in reported Pol II dynamics *in vivo*. Here we label the endogenous Pol II in mouse embryonic fibroblast (MEF) cells using the CRISPR/Cas9 gene editing system. Using single-molecule based super-resolution imaging in the living cells, we captured endogenous Pol II clusters. Consistent with previous studies, we observed that Pol II clusters were short-lived (cluster lifetime ~8 s) in living cells. Moreover, dynamic responses to serum-stimulation, and drug-mediated transcription inhibition were all in agreement with previous observations in the exogenous Pol II MEF cell line. Our findings suggest that previous exogenously tagged Pol II faithfully recapitulated the endogenous polymerase clustering dynamics in living cells, and our approach may in principle be used to directly label transcription factors for live cell imaging.

RNA Polymerase II (Pol II) is the molecular complex responsible for the synthesis of all messenger RNAs, as well as some non-coding RNAs in all eukaryotes^1^,^2^,^3^,^4^,^5^. The ability to label and image Pol II dynamically *in vivo*^6^,^7^,^8^,^9^,^10^,^11^ has resulted in new mechanistic understanding on the organization and dynamics of transcriptions. Previous approaches in mammalian cells however relied on the ability to render exogenous Pol II resistant to a toxin, α-amanitin that degrades non-mutant (endogenous) Pol II. Thus stable cell lines can be created where endogenous Pol II is degraded and replaced by labeled Pol II, often expressed under a strong exogenous promoter, and randomly inserted in the genome^10^,^12^. It was unclear then whether genome insertion context or the uncontrolled expression of exogenous Pol II may have played a significant role in the protein dynamics observed.

Here we adopted a CRISPR/Cas9 based approach for inserting fluorescent proteins at specific sites in the mammalian genome^13^,^14^,^15^,^16^,^17^. We directed Cas9 nuclease to the endogenous locus of *Rpb1*, the largest subunit of Pol II, in the N-terminus of the gene. We then designed repair templates with Dendra2 sequence flanked by left and right arms comprised of the endogenous *Rpb1* sequence context, and relied on homology-directed repair for the insertion of Dendra2 at the endogenous locus^18^. A PCR assay allows us to differentiate cells with no insertion, from cells where Dendra2 was inserted. Single-cell fluorescence-activated (FACS) cell sorting assays allowed us to select Dendra2-inserted cells from the population of cells. We investigated the organization and dynamics of the labeled, endogenous Pol II using single-molecule based super-resolution imaging^10^,^12^.

## Results

We used a recently established CRISPR/Cas9-mediated genome engineering system along with homology-directed DNA repair^15^,^16^ to fuse a fluorescent protein to one subunit of endogenous Pol II. We selected the largest subunit of Pol II, Rpb1, which is also the catalytic subunit, located on chromosome 11 in mouse genome^3^,^19^. To image with photo-activated localization microscopy (PALM)^20^, we chose to label the subunit with Dendra2, a photo-convertible fluorescent protein, akin to previous studies^10^,^12^.

**Figure 1:**
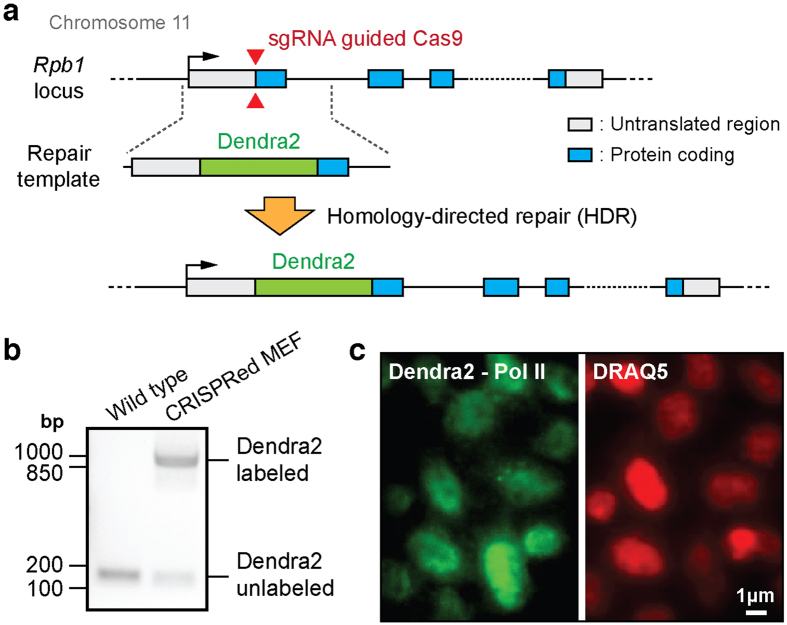
Endogenous labeling of Pol II with Dendra2 via CRISPR/Cas9-mediated knock-in in mouse embryonic fibroblasts. (**a**) Schematic illustration of CRISPR/Cas9-mediated endogenous tagging of *Rpb1* gene with Dendra2. The largest subunit of Pol II, *Rpb1* gene, is targeted for fusion with photo-convertible fluorescent protein, Dendra2. A strategy consisting of sgRNA guided Cas9 double-strand break (DSB) followed by homology-directed repair (HDR) is used. sgRNA sequence cloned Cas9 plasmids and an insertion or repair template are co-transfected in MEF (**b**) A PCR assay is designed such that without Dendra2 insertion (wild type) only a short fragment (~180 bp) is amplified. When the Dendra2 sequence is successfully inserted in at least one endogenous *Rpb1* allele, the fragment length increases to ~870 bp. Raw gel results are presented in Supplementary Fig. 3. (**c**) A pre-converted Dendra2 image under 488-nm laser illumination reveals cells with labeled endogenous Pol II. The primarily nuclear-localization of labeled endogenous Pol II (in green) is confirmed by DNA staining with DRAQ5 (in red). Together these data suggest cell lines have been successfully created with Dendra2 fused to endogenous Pol II.

Briefly about the CRISPR/Cas9 design (additional details in Methods), to induce Cas9-mediated DNA double-strand break (DSB) near the start codon of the *Rpb1* gene, the first exon and a part of the first intron were targeted with single guide RNAs (sgRNAs) that confer specificity to the Cas9 nuclease (Fig. 1a and Supplementary Fig. 1a). From sgRNA candidates, calculated from an online CRISPR Design Tool^16^, we selected three sgRNA sequences with minimal predicted off-target effects (Supplementary Table 1 and Methods). Then, each sgRNA was cloned into a *Streptococcus pyogenes* Cas9 (SpCas9) vector with a distinct promoter to co-express the sgRNAs and Cas9 *in vivo* (Supplementary Fig. 1b)^16^. A repair/fusion template was designed with the Dendra2 sequence (without a stop codon) inserted between left and right homology arms of Rpb1 (Fig. 1b and Supplementary Table 2). Silent (amino acid preserving) mutations were made at the sgRNA-targeted sequences on the repair template, to prevent digestion of the repair template by Cas9.

Among the three sgRNAs, one was successful in generating fluorescently tagged proteins as imaged on the microscope under 488-nm illumination (Supplementary Fig. 1c). We isolated the fluorescent cells using fluorescence-activated cell sorting (FACS) (Supplementary Figs 1d and 2).

We designed a PCR assay for the target locus to test for Dendra2 gene insertion. A total of 4 cell lines showing Dendra2 insertions were ultimately tested after FACS (Fig. 1b, Supplementary Figs 1e and 3). One of those cell lines was chosen at random as the final endogenous Dendra2-Pol II cell line for *in vivo* imaging of endogenous Pol II dynamics. We further verified the final cell line via sequencing, to confirm the genomic location of Dendra2 insertion and whether the integrated sequence via homology-directed repair is consistent from our repair template. Indeed the insertion was located precisely upstream of *Rpb1* gene targeted, and all three silent mutations from the repair template were recovered on the gene (Supplementary Fig. 4).

The final endogenous Dendra2-Pol II cell line was also tested on the microscope (Fig. 1c). A small percentage of unlabeled cells may remain in mixture due to imperfections in the single-cell FACS sorting, but we observe that the virtually all cells showed fluorescence under 488-nm illumination consistent with the PCR assay (Fig. 1c). As expected, Pol II was confirmed to localize in cell nucleus by simultaneous staining of DNA with a far-red intercalating dye DRAQ5^21^ (Fig. 1c).

We also tested whether the Dendra2 insertion has differentially affected gene expression as compared to the the wild type MEF. We picked expression of the β-actin gene, as it exhibits a well characterized stereotyped response to serum stimulation which can be tested with mRNA labeling. We performed fluorescent *in situ* hybridization (FISH), with Fluorescein (FITC) labeled DNA probes targeted intron 1 of β-actin gene (Supplementary Fig. 5) to label the β-actin mRNA in both wild type MEF, as a control, and the final endogenous Dendra-Pol II. Upon serum-stimulation, intensity was altered over time and peaked at 15min after serum addition for both wild type MEF, and the final endogenous Dendra2-Pol II MEF. Our results (Supplementary Fig. 5) showed good agreement in the stereotyped response of the β-actin genes, between the wild type and final Dendra2-Pol II MEF, and consistent with the previous observation with MS2-tagged β-actin mRNA in living cells^12^.

**Figure 2:**
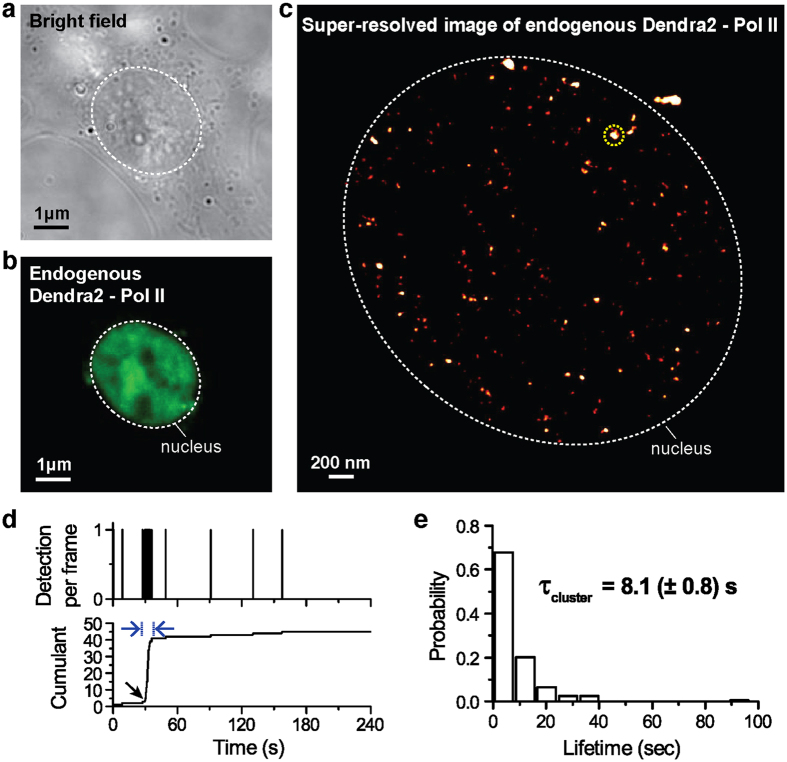
Super-resolution imaging reveals dynamic clusters of endogenously labeled Pol II in living MEF cells. (**a**) A bright field image of an CRISPR/Cas9 engineered Dendra2-Pol II live MEF cell. White dashed line indicates a nucleus. (**b**) A pre-converted Dendra2-Pol II image under illumination with 488-nm laser in the corresponding cell. Dendra2 fluorescence signals are primarily localized in the cell nucleus where Pol II activity is expected (**c**) Super-resolution reconstruction of post-converted Dendra2-Pol II, depicting endogenous Pol II clusters in the corresponding living cell. Red-hot color code is used to represents spatial density of detections (**d**) A tc-PALM analysis is performed as previously described^10^,^12^ and a representative time trace of a selected Pol II cluster (the selected example is delineated in yellow circle in (**c**). In the trace, time t = 0 represents the start of acquisition. Black arrow indicates the onset of Pol II clustering, and blue arrows indicate the apparent cluster lifetime. (**e**) A histogram of the apparent cluster lifetimes (τ_cluster_) is presented for 168 clusters from N = 13 cells. The estimated average lifetime, τ_cluster_, of endogenous Pol II cluster of 8.1 (±0.8) s was obtained. Errors (in parentheses) represent standard error of the mean.

We sought to further characterize the organization and dynamics of endogenously labeled Pol II molecules, with high resolution imaging in living cells. Firstly we imaged pre-converted Dendra2 with 488-nm illumination (Fig. 2a,b). Then we photoconverted single Dendra2 molecules with a low intensity (1.3 W/cm^2^) 405-nm laser^12^. Post-converted Dendra2 was then imaged with a 561-nm laser.

Super-resolution images of Pol II in living cells were reconstructed from 5000 frames acquired at 50 ms per frame as described previously^12^. For super-resolution reconstruction, single-molecule detections were localized with 2D-gaussian fit, and the local density of detection was represented using a red-hot color code (Fig. 2c). In the super-resolved image, we observe bright foci of endogenous Pol II clusters.

With time-correlated PALM analysis^10^,^12^, we find that the endogenous Pol II clusters are transient, consistent with the exogenous Pol II clusters from the previous studies^10^,^12^ (Fig. 2d). In the representative time trace in Fig. 2d (region selected is denoted as a yellow circle in Fig. 2c), the time profile of the number of single-molecule detections per frame is shown in the upper panel and the cumulative detection count is shown in the lower panel. At around t = 30 s after the beginning of acquisition (t = 0), endogenous Pol II signals were frequently detected for ~9 seconds. This appears as a sharp increase in slope in the cumulant graph followed by abrupt transition to a plateau, which we interpret as the rapid assembly then disassembly of a Pol II cluster. Remarkably, the average endogenous Pol II cluster lifetime of 8.1 (±0.8) s is indeed in excellent agreement with that (8.3 (±0.2) s) reported previously for exogenous Pol II clusters in live MEF cells^12^ (Fig. 2e).

**Figure 3:**
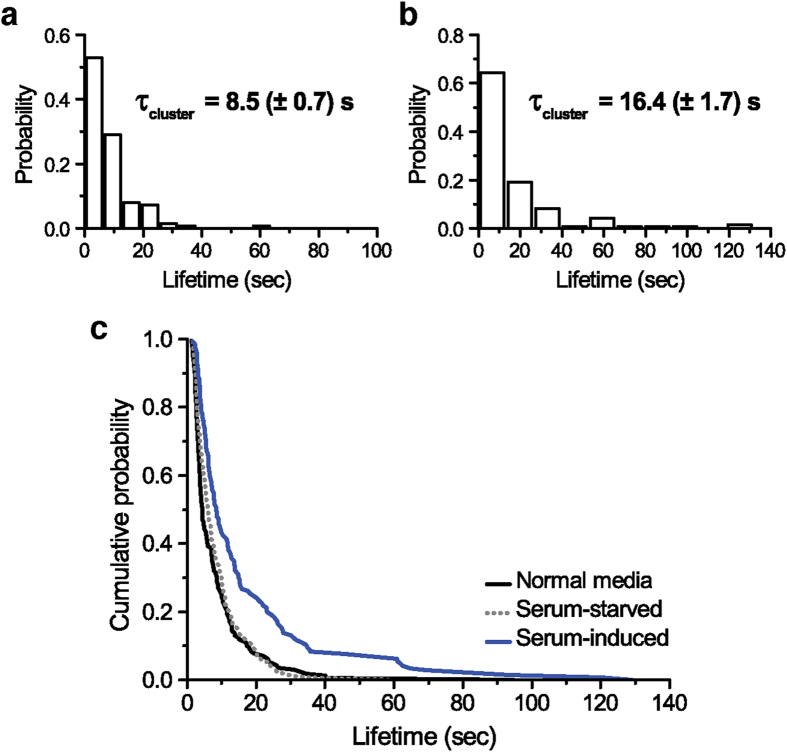
Dynamic response of endogenous Pol II cluster under serum-starvation and serum-stimulation. (**a**,**b**) Histograms of endogenous Pol II cluster lifetime distributions under serum-starvation and serum-stimulation, respectively. (**c**) The rank-ordered, Kaplan-Meier estimator (survival plots, estimated as 1 minus cumulative distribution function) is plotted to compare cluster lifetime distribution for cells grown in normal condition (black line), in serum starved (gray dashed line), or serum-induced (blue line) conditions. While cluster lifetimes in normally grown and serum-deprived MEF cells are comparable, the distribution broadens with serum-induced condition, in agreement with a previous observation that endogenous Pol II cluster lifetime may change in manner correlated with induced gene expression^12^.

Next, we tested whether endogenous Pol II clusters exhibited a dynamic response to serum stimulation. We measured Pol II cluster lifetimes under serum-starvation and serum-stimulation (Fig. 3a,b). We find that upon serum stimulation there is globally a 2-fold increase in the average Pol II cluster lifetime (16.4 (±1.7) s), comparable to the previous observation that cluster lifetime on a single gene can change between 2 and up to 4-fold on average^12^. The endogenous Pol II cluster lifetime (8.5 (±0.7) s) in the serum-starved cells did not change compared to cluster lifetime in cells grown under normal medium, confirming that the serum stimulation and not cell stress was responsible for the change in cluster lifetime during serum induction. This conclusion is further supported by survival plots of cluster lifetime (defined as ‘1 minus normalized cumulative distribution function, i.e. 1-CDF), where the frequency of long-lived clusters under serum-stimulation was higher than normal-media and serum-starved (Fig. 3c).

Finally, we tested how endogenous Pol II clusters respond to drug that inhibits transcription elongation. Previous studies suggested that disassembly of the transient Pol II cluster is stalled with drugs that inhibit entry of Pol II into the elongation phase of transcription^10^,^12^. After transcription initiation, CDK9 (cyclin-dependent kinase 9), a subunit of positive transcription elongation factor b (P-TEFb), phosphorylates Pol II to trigger entry into productive elongation^22^,^23^,^24^. Flavopiridol and DRB (5,6-Dichloro-1-beta-D-ribofuranosyl-benzimidazole) are two transcription inhibitors that block the escape of Pol II from the promoter-proximal paused state interrupting phosphorylation by CDK9^25^,^26^,^27^,^28^.

**Figure 4:**
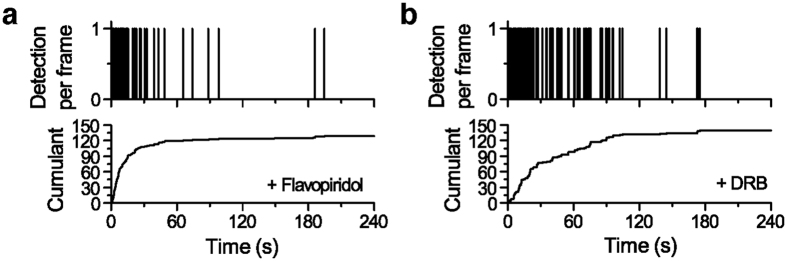
Endogenous Pol II cluster stabilize with transcription inhibitor treatment. Example time-dependent detection profiles of Pol II clusters after (**a**) 10 μM flavopridol and (**b**) 100 nM DRB treatment, respectively, show a slope onset from the beginning of aquision followed by a more gradual transition into a plateau. This signature of stable clusters under drug treatment in living cells is in stark contrasts to the transient cluster profile seen under normal conditions (e.g. illustrated in Fig. 2d). This data from endogenously tagged Pol II clusters is consistent with the previous report that Pol II cluster stability can be controlled by treatments with drugs that block promoter escape.

Upon treatments with both flavopiridol and DRB, independently, we observe that normally transient endogenous Pol II clusters become stable (Fig. 4). In the tcPALM traces, this is evident as signal detection from the beginning of acquisition followed by a gradual plateau due to photo-bleaching, as observed in the previous studies with clusters of exogenously express Pol II^10^,^12^.

## Discussion

Previously, studies requiring Pol II labeling and imaging in mammalian cells relied on degrading endogenous Pol II with a toxin, α-amanitin, and the replacement with a mutant polymerase that is resistant the toxin. As such, it was unclear what role over-expression of the exogenous transcription component may play in the reported organization and dynamics of Pol II *in vivo*. Here we report the tagging of endogenous Pol II via a CRISPR/Cas9-mediated labeling system in living cells. Consistent with what was observed in previous cell lines where labeled exogenous Pol II was imaged, we observe that endogenous Pol II clusters exist in the nucleus of living cells. The Pol II clusters were transient with a lifetime of ~8 seconds, which is in excellent agreement with previous measurements. Moreover, dynamic responses to serum-starvation, serum-stimulation and transcription inhibitors were also consistent with previous observations. Our study describes a CRISPR/Cas9 based approach which may in principle be used to directly label transcription factors for live cell imaging with high spatial and temporal resolution.

## Methods

### Cell culture

The mouse embryonic fibroblast (MEF) cells, WT SV40 MEF (ATCC CRL-2907) purchased from American Type Culture Collection (ATCC, PO Box 1549 Manassas, VA 20108 USA), were cultured in Dulbecco’s modified Eagle’s medium (DMEM) from Thermo Fisher Scientific (Cambridge, MA), supplemented with 10% fetal bovine serum (FBS) (from Thermo Fisher Scientific, 26140, qualified, US origin), 10 U/ml penicillin and 10 μg/ml streptomycin (from Thermo Fisher Scientific, 15140). The cells were grown in a water-saturated atmosphere in a 37 °C incubator containing 5% CO_2_.

### Single-guide RNA (sgRNA) cloned Cas9 plasmid

Guide RNAs were designed using the web-based CRISPR Design tool (http://crispr.mit.edu)^16^ and three sequences were selected considering off-target effects (Supplementary Fig. 1). DNA oligonucleotides of sgRNA sequences with *Bbsl* restriction sites were synthesized from Integrated DNA Technologies (IDT, Coralville, IA) (Supplementary Table 1). The oligonucleotides were cloned into *Streptococcus pyogenes* Cas9 vector (pSpCas9(BB)-2A-Puro (PX459) V2.0, #62988) from Addgene (Cambridge, MA 02139), with the *Bbsl* restriction enzyme^16^ (Supplementary Fig. 1). Constructs were transformed into *Stbl3* competent cells (Life Technologies), resistant colonies were then confirmed by sequencing. pSpCas9(BB)-2A-Puro (PX459) V2.0 was a gift from Feng Zhang (Addgene plasmid #62988).

### Homology-directed repair (HDR) DNA template

Left homology arm (LHA) and right homology arm (RHA) sequences were designed from targeted *Rpb1* gene with lengths of ~500 bp for each, from the ATG start codon of *Rpb1* (Supplementary Table 2). The Dendra2 sequence was inserted between the homology arms without the stop codon of Dendra2 to fuse Dendra2 with Rpb1. Silent mutations for sgRNA target sites were designed for the repair template to reduce Cas9 degradation of the repair template. The full repair template plasmid was synthesized using GeneArt^®^ Gene Synthesis from Life Technologies (Carlsbad, CA) and PCR-amplified to obtain linearized repair DNAs (Supplementary Table 3).

### Co-transfection of sgRNA-Cas9 plasmids and Dendra2 repair DNAs

500 ng sgRNA-Cas9 plasmids and 500 ng Dendra2 repair DNAs were chemically transfected using X-tremeGENE9 DNA Transfection Reagent from Roche Life Science (Basel, Switzerland) in MEF cells cultured in T-25 flasks with a confluency of 70%. After incubating for 24 hrs in a 37 °C incubator containing 5% CO_2_, the transfected cells were passaged and stably cultured for several passages.

### Flow cytometric cell sorting using fluorescence-activated cell sorting (FACS)

Successful CRISPR/Cas9 mediated Dendra2 inserted cells were confirmed on the microscope stage under 488-nm illumination before FACS. Initially, about 5% of cells showed fluorescence in the nucleus suggesting proper genome editing. For FACS preparation, cells were centrifuged to remove culture media, then suspended in FACS buffer (1mM EDTA, 25mM HEPES (PH 7.4), 1% FBS in 1× PBS buffer) and strained with a 35 μm sieve (from Falcon, #2235). Cells were sorted using MoFlo (from Beckman Coulter) in the Koch Institute Flow Cytometry Core at MIT. The top 5% brightest cells under 488-nm illumination were sorted in 96-well plates, with an average of one cell in each well for monoclonal amplification. Pure MEF cells (without CRISPR/Cas protocol) were used as a negative control for FACS. The sorted cells were grown in the recovery buffer (DMEM, supplemented with 30% FBS).

### PCR analysis to confirm Dendra2 gene insertion

After growing the sorted cells for 1 week in the 96-well plate without passage, six colonies in six distinct wells survived. The surviving colonies were then separately cultured as distinct cell lines in different flasks. In each cell line, we checked whether the Dendra2 gene is inserted using PCR primers for the targeted gene loci (Supplementary Table 3). Genomic DNA for each cell line was isolated using GeneElute™ Mammalian Genomic DNA Miniprep Kits from Sigma-Aldrich (St. Louis, MO). PCR products were analyzed on a 0.8% agarose gel. Four cell lines clearly showed bands in the level of Dendra2 insertion on the gel. One of the cell lines was picked for endogenous Pol II cluster experiments (Supplementary Fig. 2). The cell lines undergo regular mycoplasma testing in our laboratory at Massachusetts Institute of Technology (MIT, Cambridge, MA).

### Serum starvation and serum stimulation

Normally grown cells were split onto 25 mm round cover glass (CS-25R, from Warner Instruments, Hamden, CT) and were grown in DMEM supplemented with 10% FBS until the confluency reached 70%. The media were then exchanged with serum-free media (DMEM, 10 U/ml penicillin and 10 μg/ml streptomycin) for serum starvation. The cells were maintained in serum-free media more than 20 hrs in the 37 °C incubator before imaging for serum-starved cells. Cells were imaged in L-15 (Leibovitz) medium on the microscope. For serum-stimulation experiments, the serum-starved cells were treated with 10% FBS in L-15 medium, 10–15 min prior to live cell imaging.

### Flavopiridol and DRB treatment

Cells were plated on 25 mm round cover glass and maintained in DMEM containing 10% FBS and penicillin/streptomycin until a confluency of 70% was reached, as described above. For a flavopiridol treatment, 10 μM flavopiridol hydrochloride hydrate (from Sigma, F3055) was supplemented with L-15 medium (with 10% FBS) prior to live cell image acquisitions. For DRB (5,6-Dichloro-1-beta-D-ribofuranosyl-benzimidazole) treatment, 100 μM DRB (from Sigma, D1916) was added to L-15 medium supplemented with 10% FBS. Drug effects can be observed within minutes to tens of minute in individual cells at these concentrations, compared to several hours in lower concentrations.

### Super-resolution PALM imaging

Live cell photo-activated localization microscopy (PALM) was carried out with a Nikon Eclipse Ti microscope (from Nikon, Tokyo, Japan) with a 100× oil immersion objective lens (NA 1.40). A 488-nm laser beam (for pre-converted Dendra2 excitation), a 405-nm laser beam (for Dendra2 conversion) and a 561-nm laser beam (for post-converted Dendra2 excitation) were combined in an external platform; the combined beam was expanded and re-collimated with an achromatic beam expander (from THORLABS, Newton, NJ, AC254-040-A and AC508-300-A), and focused with an achromatic converging lens (from Edmund Optics, #45–354) into the rear focal plane of the objective lens. Images were acquired with an Andor iXon Ultra 897 EMCCD camera using Micro Manager 1.4^29^. The laser power densities used for PALM imaging were 1.3 W/cm^2^ (for 405-nm laser) and 3.2 kW/cm^2^ (for 561-nm laser) on the objective lens focused image plane.

For live cell PALM imaging of Dendra2-Pol II expressing cells, DMEM was substituted with L-15 media supplemented with 10% FBS. The cells were maintained at 37 °C on the microscope stage in a temperature controlled platform (from Invivo Scientific) during image acquisition. Image sequences were acquired with an Andor iXon Ultra 897 EMCCD camera (Andor Technology, Belfast, United Kingdom) at a rate of 50 ms per frame with 1000 EM-gain under 405-nm illumination for photo-conversion and 561-nm for post-converted Dendra2 excitation. Perfect Focus System (PFS) of the Nikon Eclipse Ti microscope was used for maintaining z-position of the microscope stage during acquisition.

### PALM image analysis

Super-resolution image analysis was performed as described before^10^,^12^,^30^. Acquired raw image data were analyzed using a custom adaptation of the multiple-target tracking algorithm (MTT)^31^ written in Matlab. The point-spread function (PSF) of temporally and spatially separated individual single fluorescence was fitted with a 2-D Gaussian distribution for localization. The center of the Gaussian fit was used as a precise estimate of the position of detected fluorophore. The localization precision was measured to be 31nm for Dendra2 in living cells^12^.

### Time-correlated PALM (tc-PALM) analysis of Pol II cluster

tcPALM analysis was performed as reported previously^12^. During live cell PALM imaging, diffusing fluorescent molecules are motion-blurred and not detectable, thus only immobile fluorescent molecules within 50 ms temporal window are presented in a tc-PALM plot like in Fig. 2d. The observed signal in a single frame (50 ms) is registered as a count of one detection. In addition, for the Dendra2 molecule that we use, only photo-converted signals are detected. The number of photo-converted Dendra2 molecules at a given time is controlled by controlling the power density of the 405-nm laser illumination. We set the power of 405-nm activation sufficiently low (1.3 W/cm^2^) so as to likely detect one molecule per frame in a Dendra2 densely accumulated region of interest (ROI). Consequently, at any given time, only a subset of all Dendra2 molecules is detected. Over a period of time, the frequency of detections represents the relative local density of protein. Higher frequency of detections (like the region of blue arrows in Fig. 2d), suggests a high local density of Dendra2-Pol II in the ROI. This appears as a sharp increase or slope in the cumulant graphs in a tc-PALM plot.

### Fluorescent *in situ* hybridization (FISH)

We designed 25 DNA FISH probes (Stellaris FISH Probes from LGC Biosearch Technologies, Novato, CA) labeled with Fluorescein (FITC), targeting intron 1 of beta-actin gene. To investigate transcriptional bursting of beta-actin under serum-stimulation, wild type MEF and endogenous Dendra2-Pol II MEF cells were grown on 25 mm round cover glasses (from Warner Instruments, CS-25R) for and starved in serum/FBS free DMEM for 12 hours. The cell were then serum-stimulated by exchanging the starving medium DMEM supplemented with 10% FBS. Cells were fixed with 4% paraformaldehyde, at time points corresponding to 5 min,10 min, 15 min, 20 min, 25 min and 30 min after serum-stimulation. For probe hybridization, we followed the Stellaris protocol. After fixation, to permeabilize the cells, the cells were incubated in 70% ethanol for 1 hr at 4 °C. After removing the ethanol with the Stellaris washing buffer, cells were incubated with 125 nM probe solution overnight at 37 °C in a humidified chamber. All sample dishes were imaged at same optical conditions and the same days for illumination consistency.

## Additional Information

**How to cite this article**: Cho, W.-K. *et al*. Super-resolution imaging of fluorescently labeled, endogenous RNA Polymerase II in living cells with CRISPR/Cas9-mediated gene editing. *Sci. Rep.* **6**, 35949; doi: 10.1038/srep35949 (2016).

**Publisher’s note:** Springer Nature remains neutral with regard to jurisdictional claims in published maps and institutional affiliations.

## Supplementary Material

Supplementary Information
