# Supplementary material for: Super-resolution imaging of fluorescently labeled, endogenous RNA Polymerase II in living cells with CRISPR/Cas9-mediated gene editing
Source: Sci Rep. 2016 Oct 26;6:35949. doi: 10.1038/srep35949 (PMC5080603; doi:10.1038/srep35949)
Supplement: Supplementary Information [file srep35949-s1.pdf]

Supplementary Information

Super-resolution imaging of fluorescently labeled, endogenous RNA Polymerase II in living cells with CRISPR/Cas9-mediated gene editing

Won-Ki Cho[1], Namrata Jayanth[1], Susan Mullen[1], Tzer Han Tan[1], Yoon J. Jung[1] and Ibrahim I. Cissé[1]*

[1]Department of Physics, Massachusetts Institute of Technology, 77 Massachusetts Avenue, Cambridge, MA 02139

*Correspondence to: icisse@mit.edu

Supplementary Table 1: sgRNA sequences targeted *Rpb1* gene

	Sequence (5′ ⇒ 3′)
sgRNA #1	(forward) CACCGCTCTCTTGATGGTGCGCAGC
	(reverse) AAACGCTGCGCACCATCAAGAGAGC
sgRNA #2	(forward) CACCGAGTCCTGAGTCCGGATGAAT
	(reverse) AAACATTCATCCGGACTCAGGACTC
sgRNA #3	(forward) CACCGCGGGCATGCGCTGTCCCCGG
	(reverse) AAACCCGGGGACAGCGCATGCCCGC

* Red : BbsI restricted DNA overhang sequences

Supplementary Table 2: PCR primers for repair DNA amplification and Dendra2 gene knock-in confirmation

Homology left arm<br>(5′ UTR of *Rpb1*)	CTCACTATAGGGAGACCCAAGCTGGCTAGCGTTTAAACTTAAGCTTCCTCAGAAGCGCCG<br>AGAGCGCGACCGGGACGGTTGGAGAAGAAGGTGGCTCCCGGAAGGGGGAGAGACAAACTG<br>CCGTAACCTCTGCCGTTCAGGATCCCGGTTACTTATTTATTCGTTACCCCTTTTCTTCTT<br>CCTCCCTCCCAAAACCTCCTCCTCTCCTCCCTTCTTTTGTTTCCTTTTTGGGAGCCGACG<br>AATCTCCGGAAAGGGAGAAAAGGCTTTCTTTCAGCCCTTTTCGTTCCTCTGCCTTCCCCC<br>CCCCTTCCCCCCTCCCCCACCTTTCCCTCCTCCAGCCTTTCCCTCCCTATCCCGGGAGGT<br>CTTTCCTGGTGGCCGCCCGGACGGGTTCTGAGCACTTAGGCGGCGGTGGCGCAGGCTTTT<br>TGTAGCGAGGTTTGCGCCTGCGCAGCGCGCCTGCCTCGCCATGAACACCCCGGGAATTAA
Dendra2 sequence	CCTGATCAAGGAGGACATGCGCGTGAAGGTGCACATGGAGGGCAACGTGAACGGCCACGC<br>CTTCGTGATCGAGGGCGAGGGCAAGGGCAAGCCCTACGAGGGCACCCAGACCGCCAACCT<br>GACCGTGAAGGAGGGCGCCCCCCTGCCCTTCAGCTACGACATCCTGACCACCGCCGTGCA<br>CTACGGCAACCGGGTGTTCACCAAGTACCCCGAGGACATCCCCGACTACTTCAAGCAGAG<br>CTTCCCCGAGGGCTACAGCTGGGAGCGCACCATGACCTTCGAGGACAAGGGCATCTGCAC<br>CATCCGCAGCGACATCAGCCTGGAGGGCGACTGCTTCTTCCAGAACGTGCGCTTCAAGGG<br>CACCAACTTCCCCCCCAACGGCCCCGTGATGCAGAAGAAGACCCTGAAGTGGGAGCCCAG<br>CACCGAGAAGCTGCACGTGCGCGACGGCCTGCTGGTGGGCAACATCAACATGGCCCTGCT<br>GCTGGAGGGCGGCGGCCACTACCTGTGCGACTTCAAGACCACCTACAAGGCCAAGAAGGT<br>GGTGCAGCTGCCCGACGCCCACTTCGTGGACCACCGCATCGAGATCCTGGGCAACGACAG<br>CGACTACAACAAGGTGAAGCTGTACGAGCACGCCGTGGCCCGCTACAGCCCCCTGCCCAG
Homology right arm<br>(*Rpb1* gene sequence)	CCAGGTGTGGATGCACGGGGGTGGCCCCCCCTCCGG**C**GACAGCGCATGCCCGCT**C**CGCAC<br>CATCAAGAGAGTGCAGTTCGGAGTCCTGAG**C**CCGGATGAATTGGTAAGATAGTCCGCCCG<br>TCTCTCTGCTATGCCTTTGGCTGATGATCCTCTGGGAAGATGGGTCAGTGATGCTGATGT<br>TGTGCGGGGGTCCAGCCTTCAAAAGTTAAATTATGCCAGGACTTAAACAAGCGAAATGCT<br>ATGCCTGAGATGAGTGCTTGACTGGACAGAGAAGAAAGATGTTTCCATACTTGAGCATTG<br>CTGCGGTTCAACGAGACAGGGTTGAAAGTTGTACAGGAAGTGGATCCCCAGTTTTAGTTC<br>TTTCACCAATATTGAGCATCCTGCTATGTGACAGTAATCAGACACTAGGAACACAGCTGC<br>ACAATAAATATAACAGGAATTC

* Green block : start site of Dendra2 * Yellow block : start site of *Rpb1* * **Red bolded letters** : silent mutations

Supplementary Table 3: PCR primers for repair DNA amplification and Dendra2 gene knock-in confirmation

PCR primers	Sequence (5′ ⇒ 3′)
Dendra2 homology-directed repair DNA amplification	(forward) CAGCCAGTTCTCTCCTCAGAAGCG
	(reverse) CAACCCTGTCTCGTTGAACCGCAGC
Dendra2 gene knock-in confirmation	(forward) CTATCCCGGGAGGTCTTTCCTGGT
	(reverse) TGCACTCTCTTGATGGTGCGC

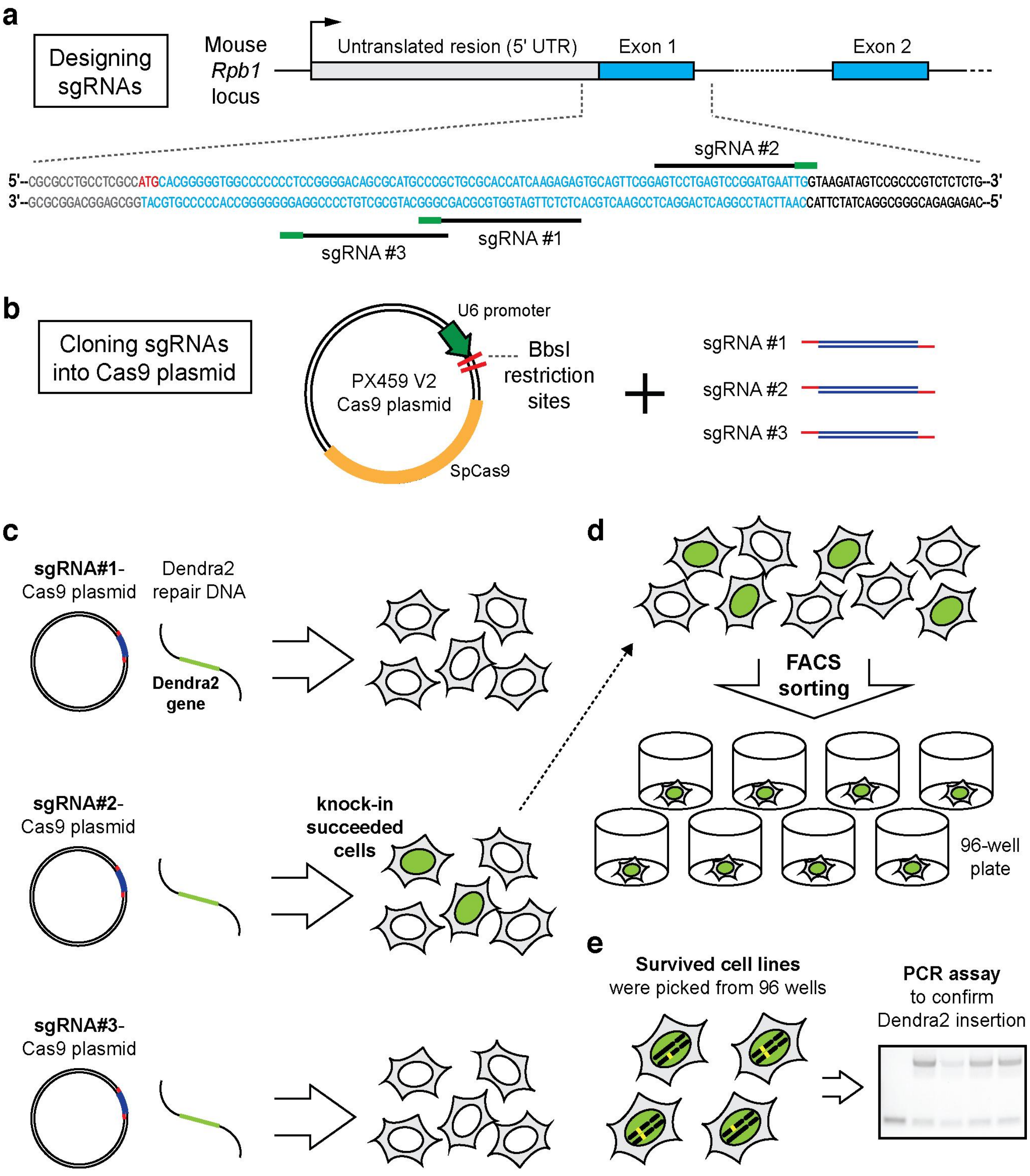

Supplementary Figure 1. Schematic procedure of CRISPR/Cas9-mediated Dendra2 labeling of RNA polymerase II

(**a**) Designing sgRNAs in the target region minimizing off-target effect. Green bars in sgRNA represent PAM sequences. (**b**) Cloning each sgRNA into Cas9 expression vector with BbsI restriction enzyme digest. (**c**) Co-transfection of sgRNA vectors along with Dendra2 repair template to living MEF cells. (**d**) Sorting of fluorescent cells in 96-well plates using FACS. (**e**) PCR assay to confirm a target gene insertion.

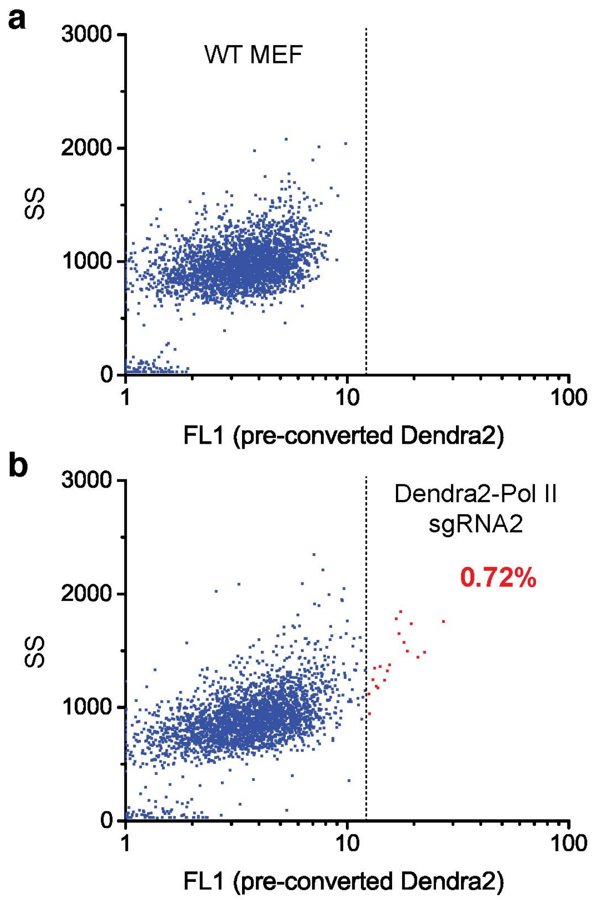

Supplementary Figure 2. Fluorescence activated cell sorting (FACS) dot plots

Cells transfected with sgRNA#2-Cas9 construct along with Dendra2 repair template were sorted using FACS to identify fluorescent cells. (**a**) A dot plot shows measurements of side scatter (SS) and fluorescence detection through FITC filter and 488-nm excitation (FL1) for 11,693 wild type cells. (**b**) A dot plot shows side scattering (SS) and fluorescence intensity of pre-converted Dendra2 for 15,888 sgRNA#2-Cas9 transfected cells. We set a sorting threshold on the maximum intensity detected in wild type cells. 0.72% fluorescent cells (N=116 cells) with intensities above the threshold were collected.

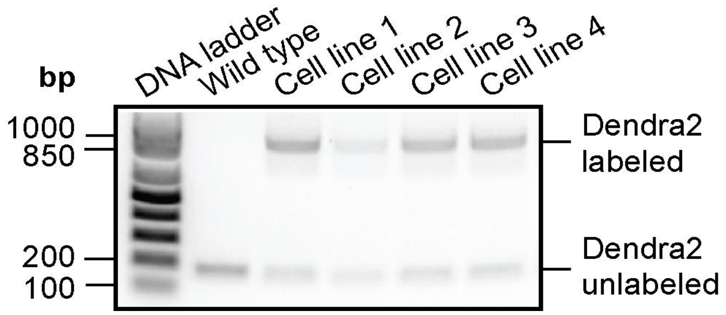

Supplementary Figure 3. PCR analysis confirms Dendra2 gene insertion

PCR primers amplify ~180bp of endogenous *Rpb1* gene around the target region. When Dendra2 gene (~690bp) is inserted properly the product of PCR should be ~870bp. In the result of PCR assay, FACS sorted cell lines show Dendra2-inserted bands unlike the wild type MEF, those are good to use for endogenous Pol II imaging.

a

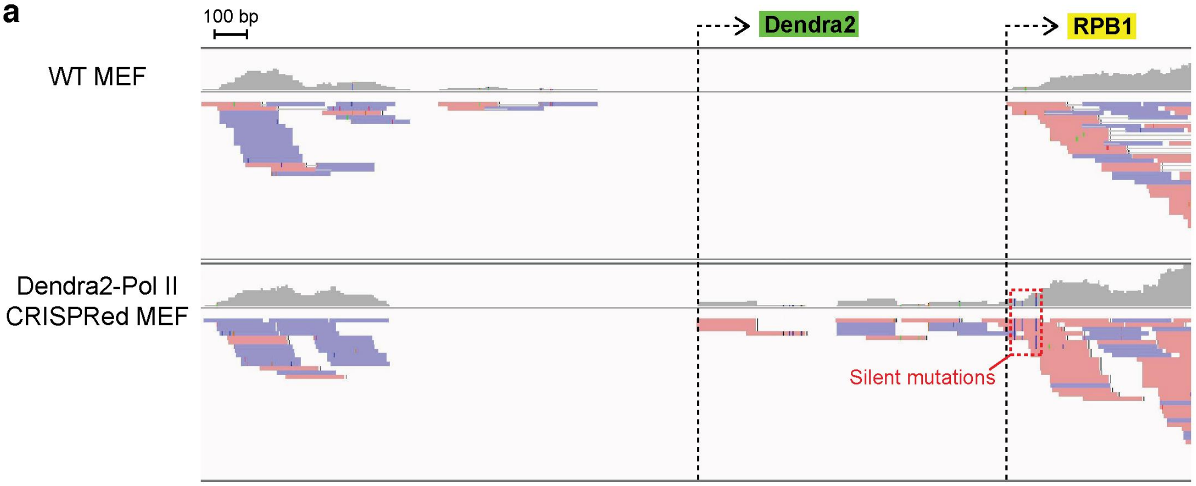

b

Sequnecing result of Dendra2-Pol II CRISPR-MEF

5'- TGC CTT CCC CCC CCC TTC CCC CCT CCC CCA CCT TTC CCT CCT CCA GCC TTT CCC TCC
CTA TCC CGG GAG GTC TTT CCT GGT GGC CGC CCG GAC GGG TTC TGA GCA CTT AGG CGG CGG
TGG CGC AGG CTT TTT GTA GCG AGG TTT GCG CCT GCG CAG CGC GCC TGC CTC GCC ATG AAC
ACC CCG GGA ATT AAC CTG ATC AAG GAG GAC ATG CGC GTG AAG GTG CAC ATG GAG GGC AAC
GTG AAC GGC CAC GCC TTC GTG ATC GAG GGC GAG GGC AAG GGC AAG CCC TAC GAG GGC ACC
CAG ACC GCC AAC CTG ACC GTG AAG GAG GGC GCC CCC CTG CCC TTC AGC TAC GAC ATC CTG
ACC ACC GCC GTG CAC TAC GGC AAC CGG GTG TTC ACC AAG TAC CCC GAG GAC ATC CCC GAC
TAC TTC AAG CAG AGC TTC CCC GAG GGC TAC AGC TGG GAG CGC ACC ATG ACC TTC GAG GAC
AAG GGC ATC TGC ACC ATC CGC AGC GAC ATC AGC CTG GAG GGC GAC TGC TTC TTC CAG AAC
GTG CGC TTC AAG GGC ACC AAC TTC CCC CCC AAC GGC CCC GTG ATG CAG AAG AAG ACC CTG
AAG TGG GAG CCC AGC ACC GAG AAG CTG CAC GTG CGC GAC GGC CTG CTG GTG GGC AAC ATC
AAC ATG GCC CTG CTG CTG GAG GGC GGC GGC CAC TAC CTG TGC GAC TTC AAG ACC ACC TAC
AAG GCC AAG AAG GTG GTG CAG CTG CCC GAC GCC CAC TTC GTG GAC CAC CGC ATC GAG ATC
CTG GGC AAC GAC AGC GAC TAC AAC AAG GTG AAG CTG TAC GAG CAC GCC GTG GCC CGC TAC
AGC CCC CTG CCC AGC CAG GTG TGG ATG CAC GGG GGT GGC CCC CCC TCC GG**C** GAC AGC GCA
TGC CCG CT**C** CGC ACC ATC AAG AGA GTG CAG TTC GGA GTC CTG AG**C** CCG GAT GAA TTG -3'

Supplementary Figure 4. Sequencing confirms integration of Dendra2 in the target locus

(**a**) Reads of Dendra2 sequences (green start site), upstream of *Rpb1* gene (yellow start site), are only detected in CRISPRed MEF and not in wild type MEF. Remarkably, silent mutations (red dashed box) that we input on the repair template, were fully confirmed on the exon 1 of *Rpb1* only in CRISPRed cell line, and not in wild type MEF. The corresponding sequencing result for the region in CRISPRed MEF is represented in panel (**b**). The sequence is perfectly conserved from that expected of our repair template (see **Supplementary Table 2**) after genome integration. We note that we also checked copy number variation (CNV) for WT MEF and CRISPRed MEF since that The SV40 immortalized cell lines used are inherently prone to chromosomal instability. For chromosome 11 where the RPB1 gene is located, ploidy was normal in wild type, while in the CRISPRed MEF loss of chromosome occurred in some cells, likely in a sub-population of the initially sorted monoclonal cells. This suggests that even in an initially monoclonal homozygous cell line, genome instability resulting from cellular immortalization may lead to loss of genomic label in a subpopulation of cells.

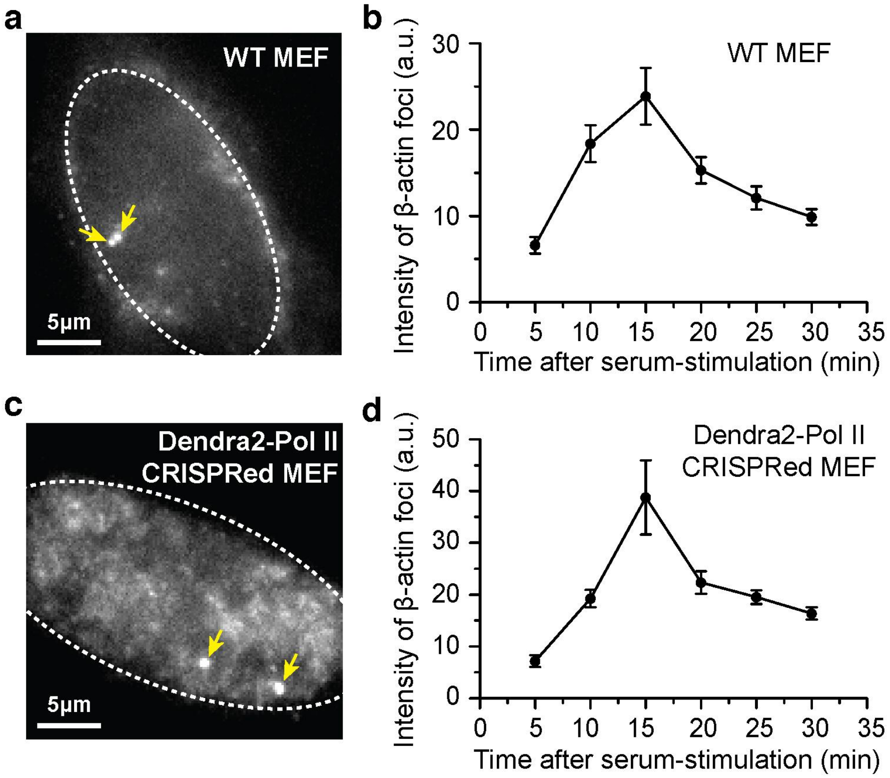

Supplementary Figure 5. Fluorescent In Situ Hybridization (FISH) for β-actin gene

(**a**) A representative image of a fixed wild type MEF cell with FISH. Yellow arrows indicate fluorescence signals of fluorescein (FITC) labeled FISH probes at β-actin gene loci under 488-nm illumination. (**b**) Intensity profile of β-actin gene foci over time after serum-stimulation in fixed wild type MEF cells. (**c**) A representative image of a fixed Dendra2-Pol II CRISPRed MEF cell with FISH. Yellow arrows indicate fluorescence signals of fluorescein (FITC) labeled FISH probes at β-actin gene loci under 488-nm illumination. Note that background of nucleus in CRISPRed cells is higher than wild type because of pre-converted Dendra2 excitation under 488-nm illumination. (**d**) Intensity profile of β-actin gene foci over time after serum-stimulation in Dendra2-Pol II CRISPRed cells. Both results, for wild type and CRISPRed cell lines, of transcription bursting for β-actin gene are in a good agreement with previous observation with MS2-tagged β-actin mRNA in living cells[1,2]. This implies that transcription function of Dendra2-labeled RNA-Pol II in CRISPRed cell line is normal.

Supplementary References

1 Cho, W. K. *et al.* RNA Polymerase II cluster dynamics predict mRNA output in living cells. *Elife* **5**, doi:10.7554/eLife.13617 (2016).

2 Lionnet, T. *et al.* A transgenic mouse for in vivo detection of endogenous labeled mRNA. *Nat Methods* **8**, 165-170, doi:10.1038/nmeth.1551 (2011).
